# Growth arrest-specific gene 6 expression in human breast cancer

**DOI:** 10.1038/sj.bjc.6604260

**Published:** 2008-02-19

**Authors:** O Mc Cormack, W Y Chung, P Fitzpatrick, F Cooke, B Flynn, M Harrison, E Fox, E Gallagher, A Mc Goldrick, P A Dervan, A Mc Cann, M J Kerin

**Affiliations:** 1 UCD School of Medicine and Medical Science, UCD Conway Institute, Belfield, Dublin 4, Ireland; 2 UCD School of Public Health and Population Science, Belfield, Dublin 4, Ireland; 3 Department of Pathology, Mater Misericordiae Hospital, Eccles St, Dublin 7, Ireland; 4 Clinical Science Institute, University College Hospital, Galway, Ireland

**Keywords:** growth arrest-specific gene 6, Axl tyrosine kinase receptor, progesterone receptor B, breast cancer

## Abstract

Growth arrest-specific gene 6 (Gas6), identified in 1995, acts as the ligand to the Axl/Tyro3 family of tyrosine kinase receptors and exerts mitogenic activity when bound to these receptors. Overexpression of the Axl/Tyro3 receptor family has been found in breast, ovarian and lung tumours. Gas6 is upregulated 23-fold by progesterone acting through the progesterone receptor B (PRB). Recently, Gas6 has been shown to be a target for overexpression and amplification in breast cancer. Quantitative real-time PCR analysis was used to determine the levels of Gas6 mRNA expression in 49 primary breast carcinomas. Expression of PRB protein was evaluated immunohistochemically with a commercially available PRB antibody. The results showed a positive association between PRB protein and Gas6 mRNA levels (*P*=0.04). Gas6 correlated positively with a number of favourable prognostic variables including lymph node negativity (*P*=0.0002), younger age at diagnosis (*P*=0.04), smaller size of tumours (*P*=0.02), low Nottingham prognostic index scores (*P*=0.03) and low nuclear morphology (*P*=0.03). This study verifies for the first time the association between PRB and Gas6 in breast cancer tissue.

In 1993, the growth arrest-specific gene 6 (Gas6) was first described (Manfioletti *et al*, 1993) and, in 1995, human Gas6 was mapped to chromosome 13q34 by fluorescence *in situ* hybridisation (Saccone *et al*, 1995). The Gas6 protein has 44% identity to human protein S, a vitamin K-dependent negative coregulator in the blood coagulation pathway. However, Gas6 lacks the thrombin cleavage site, typical of vitamin K-dependent coagulation factors. Similar to protein S, Gas6 is composed of defined structural motifs comprised of a gamma-carboxylated amino terminus (Gla domain), four tandem repeats of epidermal growth factor (EGF)-like domains and a large carboxy-terminal domain with similarity to the sex hormone-binding globulin (SHBG) (Manfioletti *et al*, 1993).

Gas6 has been identified as a ligand for the Axl/Tyro3 family of receptor tyrosine kinases (RTK), including Axl, Tyro3 (also known as Sky) and Mer (Varnum *et al*, 1995). Gas6 binds to these receptors with binding affinities in the order of Axl>Tyro3>Mer. Once bound, Gas6 induces receptor phosphorylation in a dose-responsive manner (Chen *et al*, 1997).

In murine cell lines, recombinant human Gas6 protein interacts with the extracellular domain of the Axl-RTK, leading to either increased receptor kinase activity and activation of the mitogen-activated protein (MAP) kinase pathway (Bellosta *et al*, 1997), or the phosphatidyl-inositol (PI)3-kinase pathway. Gas6 thereby exerts mitogenic activity in cell lines, and furthermore has been shown to initiate coordinated entry into the S phase of the cell cycle, when bound to endogenous Axl (Goruppi *et al*, 1996).

Overexpression of receptors for Gas6 belonging to the Axl/Tyro3 family had been reported in human mammary tumours, leukaemia and lung cancers (Crosier *et al*, 1995; Berclaz *et al*, 2001; Shieh *et al*, 2005).

Mouse studies have shown that Gas6 is a platelet response amplifier that plays a significant role in pathological thrombosis, and that Gas6-neutralising antibodies inhibit platelet aggregation *in vitro* (Angelillo-Scherrer *et al*, 2001). Furthermore, when the Gas6 receptor is blocked, initial platelet aggregation does occur, but stabilisation of platelet aggregates is impaired and mice are protected against life-threatening thrombosis (Angelillo-Scherrer *et al*, 2005). This inhibition preventing thrombus does not increase bleeding, and so makes Gas6 inhibition an ideal therapeutic option in thrombotic disorders. Recent publications have confirmed that all three RTKs (Axl, Tyro3 and Mer) are present on human platelets and that Gas6 expression is high in blood (Gould *et al*, 2005; Balogh *et al*, 2005).

In breast cancer cell lines, Gas6 has been demonstrated to be upregulated greater than 23-fold by progesterone acting through the progesterone receptor B (PRB) (Richer *et al*, 2002). In 2001, it was shown that overexpression of Gas6 resulted in the stabilisation of *β*-catenin, an E-cadherin-anchoring protein, in cultured mammalian cell lines (Goruppi *et al*, 2001). This has implications for the development of features of the epithelial-mesenchymal transition (EMT) phenotype, a well-recognised phenomenon resulting in increased tumour invasiveness, proliferation and cancer cell motility. Membrane staining of the receptor for Gas6, Axl, has been reported to be higher in cancerous tissue than in the normal breast (Berclaz *et al*, 2001). Moreover, the Gas6 locus has recently been described as a target for amplification in mouse models of breast cancer and in human breast cancer (Abba *et al*, 2007).

The current study was undertaken to validate expression of Gas6 in primary breast carcinomas and to evaluate the clinical relevance of this PRB-regulated gene in breast carcinogenesis. So far, this association has not been investigated in human breast tissue. To this end, we determined the mRNA expression levels of Gas6 in primary breast tumours using quantitative real-time PCR, correlated PRB protein and Gas6 mRNA levels in this cohort and examined the relationship of Gas6 to a number of clinical variables (eg risk factors, surgical treatment and histopathological profiles) and clinical outcome.

## MATERIALS AND METHODS

### Ethical issues and details of the tissue cohort used in this study

Fresh human breast tumour surgical specimens were collected prospectively in the Mater Misericordiae and Mater Private Hospitals (Dublin, Ireland). The experiments were carried out with the approval of the Mater Hospital Ethics Committee, and all patients participating in this study gave full and informed consent. Samples were taken from mastectomy or wide local excision surgical specimens under the supervision of a pathologist. Fresh tissue samples were snap frozen and stored at −70°C. These specimens were divided into two, one for RNA extraction and one for fixing and paraffin embedding to allow haematoxylin- and eosin-stained histological examination. There were a total of 49 specimens from patients with primary breast carcinomas. Clinical information was collected on all patients who donated a sample of tissue for the study. The pathologist associated with the study reviewed all the cases and evaluated independently a number of histological characteristics of the tissue specimens including grading subtypes (mitotic index, tubule formation, nuclear pleomorphism), presence of lymphocytic infiltration and presence of pushing or infiltrating margins. Follow-up data from outpatient clinic assessments and general practitioners were recorded and a numerically coded database was then constructed for the purpose of statistical analysis. Table 1 shows the clinicopathological characteristics of the cohort.

### Extraction of total RNA and random hexamer-primed cDNA synthesis

Total RNA was extracted from the frozen tissues using Trizol™ (MRC Inc., Cincinnati, OH, USA) according to the manufacturer's instructions. RNA concentrations were estimated from spectrophotometer absorbance readings of 260 and 280 nm. Samples were subjected to DNase treatment (Invitrogen Life Technologies Inc., Renfrew, Strathclyde, UK) to eliminate any possible DNA contamination. Single-stranded cDNA was subsequently generated using random hexamer-primed sequences and Superscript™ II (Invitrogen Life Technologies Inc.) methodology. cDNA was synthesised at 42°C for 2 h followed by inactivation of the enzyme at 70°C for 15 min. Negative controls with the omission of the reverse transcriptase were set up for all samples. Resultant cDNA samples were stored at −20°C.

### Real-time LightCycler™ quantitative Gas6 PCR

LightCycler™ real-time PCR was used to quantify Gas6. Ten microlitres of master SYBR Green (Qiagen, Crawley, West Sussex, UK) was mixed with 6 *μ*l of RNase-free H_2_O, 1 *μ*l of each primer and 2 *μ*l of cDNA template and subsequently loaded into each capillary. All quantitative assays were based on serially diluted solutions of target-specific PCR product for Gas6 and endogenous 18S rRNA (*Taqman*™ Ribosomal RNA Control Reagents; Applied Biosystems, Foster City, CA, USA). Primer sequences for 18S were obtained from on-line primer design software Primer3design (http://frodo.wi.mit.edu) (18S FWD 5′-AGGGTTCGATTCCGGAG-3′, 18S REV 5′-ACCAGACTTGCCCTCC-3′) and those for Gas6 were designed with Quantiprobe design software (www.qiagen.com/goto/assays) (Gas6 FWD 5′-GTAGCTTCCACTGTTCCT-3′, Gas6 REV 5′-GCGCACTCGTCTATGTCTT-3′). Cycling conditions for Gas6 were 95°C for 15 min followed by 40 cycles of 94°C for 15 s, 58°C for 20 s and 72°C for 12 s.

Each experiment was set up using a standard curve, with each quantitative point represented in triplicate. Test cDNA expression levels were generated from the standard curve using the in-built LightCycler™ second derivative maximum software for quantification. Quantitative normalisation of Gas6 in each sample was performed using endogenous 18S rRNA as an internal control. Subsequently, Gas6 mRNA levels are shown as amol per 1 ng endogenous 18S rRNA from the median of triplicate samples.

### PRB immunohistochemistry

Serial 5 *μ*m sections were prepared from each of the corresponding formalin-fixed paraffin-embedded tumour blocks that had been identified pathologically. Immunohistochemical evaluation of the PRB protein was carried out using the commercially available lyophilised monoclonal antibody (NCL-PGR-B) clone SAN27 from Novocastra, (Newcastle Upon Tyne, UK) with antigen retrieval using Trilogy (20 × ). The primary antibody, PRB, was applied. A biotinylated secondary antibody was then added, followed by an avidin and biotinylated horseradish peroxidase macromolecular complex (Vectastain Universal Elite ABC kit, Vector Laboratories, Burlingame, CA, USA). Following application of the chromagen (ChemMate Dako Envision™ Kit), sections were counterstained and quantified. A positive result was one where >10% of the cells exhibited nuclear staining.

### Statistical analyses

SAS version 8 (SAS Institute Inc., Cary, NC, USA) was used for all statistical analyses. The *χ*^2^ test was used for comparison of proportions, correlation coefficients were calculated to determine the strength of the relationship between two continuous variables and the *t*-test, Wilcoxon rank sum test or analysis of variance was used as appropriate to compare groups. Logistic regression was performed to adjust for confounders, where the outcome variable was binary. Multiple regression was carried out to adjust for confounders where the outcome variable was continuous. Initially, all of the standard clinicopathological variables were looked at in univariate analyses to identify those variables that were significantly associated with disease-free interval (DFI) and overall survival (OS). In addition, the laboratory results for Gas6 mRNA were similarly analysed. The clinicopathological and laboratory variables found to be significant in the univariate analyses (lymph node status, ER*α*, stage, CEA levels and CA 15.3 levels for DFI; (lymph node status, Nottingham prognostic index (NPI), stage, CA 15.3, size and margins for OS) were subsequently used in the Cox multivariate model to identify those with independent significance for both the DFI and OS outcomes.

## RESULTS

### Quantitative real-time PCR results for Gas6

**Figure 1:**
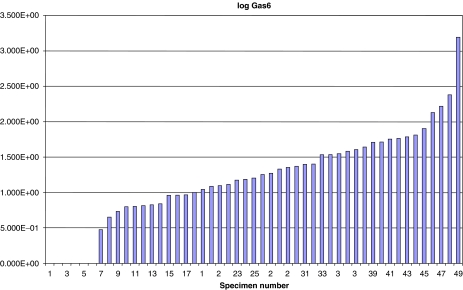
Quantitative real-time PCR expression levels of Gas6 in primary breast carcinomas.

The resultant ratios of Gas6 mRNA to endogenous ribosomal 18S mRNA are shown in Figure 1. All the specimens were positive for ribosomal 18S; however, six samples were negative for Gas6 mRNA expression and there was a wide variation in the Gas6 mRNA levels in the primary tumour cohort.

### Association of Gas6 results with PRB profiles

When the Gas6 mRNA results were examined in relation to the PRB immunohistochemical profiles, there was a positive association between PRB immunohistochemistry (positive>10%) and Gas6 mRNA levels in curatively resected cancers (stage 4 disease was omitted) (Table 2).

### Comparison of Gas6 results with clinical variables

Gas6 mRNA expression was inversely associated with the presence of lymph node metastases. The majority of younger women (80%) had high Gas6 mRNA levels, whereas the majority of older women (56%) expressed low levels of Gas6 mRNA. There was a significant inverse association between tumour size and Gas6 expression. Larger tumours (average size=38 mm) were associated with low Gas6 levels and smaller tumours (average size=22 mm) were associated with high Gas6 levels. When Gas6 expression was compared to the NPI, there was an inverse correlation. Tumours with low Gas6 levels were found to have high NPI scores (average=5.1, poor prognosis) and those with high Gas6 expression had low NPI scores (average=3.94, moderate 1 prognosis). This association held true when adjusted for confounding variables such as tumour size. In the low Gas6 group, 90.3% of the samples had a nuclear morphology of 3. The tumour group with low Gas6 levels was found to have infiltrating margins on histology, whereas the group with high Gas6 levels was found to have pushing margins (Table 2).

**Figure 2:**
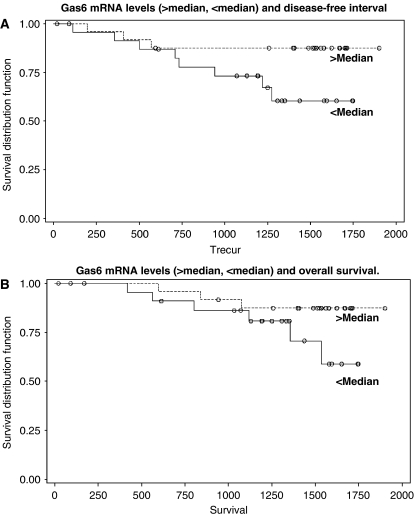
Kaplan–Meier curves. (**A**) Gas6 mRNA levels (>median, <median) and DFI. (**B**) Gas6 mRNA levels (>median, <median) and OS.

The mean duration of follow-up was 3.5 years, with a range of 21 days to 5.2 years. With regard to DFI, 12 patients (24%) developed a recurrence during the follow-up period of this cohort. At the end of the follow-up, 36 (74%) were alive and well, 3 (6%) were alive with breast cancer recurrence, 9 (18%) had died from breast cancer and 1 person (2%) had died from another cause. Gas6 mRNA levels were not independently associated with DFI or OS in this cohort by univariate or multivariate analysis (Figure 2A and B). Significant variables for DFI included lymph node metastases (*P*=0.02), ER*α* immunohistochemistry (*P*=0.03), stage (*P*=0.003), CEA (*P*=0.01) and CA 15.3 (*P*=0.05) (Table 3). The variable that remained significant for DFI after multivariate analysis was stage. Other variables were nonsignificant. In the survival analyses, significant variables were lymph node status (*P*=0.01), NPI (*P*=0.003), stage (*P*=0.002), CA 15.3 (*P*=0.05), tumour size (*P*=0.04) and tumour margins (*P*=0.05) (Table 4). Similar to DFI, stage was the only independent predictor of OS in this analysis.

## DISCUSSION

Gas6 expression correlated significantly with PRB expression in this cohort (*P*=0.04). This is the first documented positive relationship between PRB and Gas6 in human breast tumours and extends the previous work of Richer *et al* (2002) on breast cancer cell line. Gas6 has displayed mitogenic activity in cell lines; however, in our cohort, Gas6 was associated with favourable prognostic markers including smaller tumour size (*P*=0.02), decreased levels of lymph node metastases (*P*=0.0002) and lower nuclear morphology (*P*=0.03). Gas6-positive tumours were associated with pushing margins on histological assessment. Pushing margins have been found in BRCA1 mutation-positive patients with breast cancer (Lakhani, 1999). Interestingly, a recent publication has cited an association between BRCA1 mutation and increased progesterone receptor signalling (Ma *et al*, 2006). This would be in keeping with our findings.

As the association between PRB and Gas6 had not been validated previously, this study has the potential to generate further work in this area. At present, the only commercially available antibodies for growth arrest-specific proteins (including Gas1 and Gas6) are polyclonal (produced by Santa Cruz Biotechnology Inc.). To date, specificity of this antibody has been problematic on western blot analysis.

Gas6 is an attractive target for molecular manipulation, as it is the ligand of the Axl tyrosine kinase receptor, which has previously been shown to be overexpressed in a subset of breast carcinomas (Berclaz *et al*, 2001). Moreover, expression of both Gas6 and Axl is increased in ovarian, endometrial and prostate carcinomas (Sun *et al*, 2003, 2004; Sainaghi *et al*, 2005), confirming expression of these potential targets in hormone-responsive tumours.

Studies focusing on the role of Gas6 in coagulation pathways have shown that Gas6 is a platelet response amplifier that plays a significant role in pathological thrombosis. These studies have progressed and inhibition of Gas6 or the Axl receptor has been achieved in mouse models. When the receptor is blocked, initial platelet aggregation does occur, but stabilisation of platelet aggregates is impaired and mice are protected against life-threatening thrombosis (Angelillo-Scherrer *et al*, 2005). Notably, when Gas6 was inhibited, thrombus was prevented; however, there was no increase in bleeding. This makes Gas6 inhibition an ideal therapeutic option for thrombosis.

Similar to most cancer patients, breast cancer patients are at an increased risk of developing thrombosis. In addition, there is an increased risk of thrombo-embolic events such as stroke, pulmonary embolism and deep vein thrombosis, when using tamoxifen with cytotoxics. Hypothetically, an agent that targets Gas6 or Axl would benefit women with breast cancer by blocking a downstream effector of progesterone and potentially decreasing thrombosis, without causing increased bleeding. Theoretically, such a strategy could be used with conventional anti-oestrogen agents such as tamoxifen and aromatase inhibitors.

Further work examining the role of Gas6 as an anti-hormone treatment in breast cancer with the additional characteristic of it preventing thrombosis is an attractive area for future investigations.

## Figures and Tables

**Table 1:** Histopathological profiles of patients in the Gas6 study

**Patient attribute**	**Number**	**Percentage**
Total primary breast cancers	49	100
Mean age (range)	60 years (33–87 years)	
		
*Cancer type*		
Invasive ductal	42	86
Invasive lobular	1	2
Mixed	6	12
		
*Tumour size*		
<20 mm	4	8
20–50 mm	33	67
>50 mm	12	25
		
*Tumour grade* (Elston and Ellis, 1991)		
I	1	2
II	28	57
III	20	41
		
*Lymph node status*		
0	19	39
1–3	15	30.5
>3	15	30.5
		
*Nottingham prognostic index (*Blamey, 1996)		
<2.4, excellent	0	0
2.5–3.4, good	5	10
3.5–4.4, moderate I	13	27
4.5–5.4, moderate II	11	22
>5.4, poor	20	41
		
*HER-2/neu status*		
Positive	3	6
Negative	13	27
Unknown	33	67

**Table 2:** Clinical variables statistically associated with Gas6

		**Gas6 mRNA**		
**Variable**		**<2.2 × 10^6^**	**>2.2 × 10^6^**	***P*-value**
*PRB IHC (n=30)*				
Negative (⩽10%)		10 (77%)	3 (23%)	0.04
Positive (>10%)		7 (35%)	11 (65%)	
				
		**<Mean**	**>Mean**	
*Lymph node metastases (n=49)*				
Average (s.d.)		4.219 (5.35)	0.5 (1.07)	0.0002
(s.e.m.)		(0.84)	(0.378)	
				
		**<Mean**	**>Median**	
*Nuclear morphology (NM) (n=38)*				
NM=1 or 2 (*n*=7)		4 (57%)	3 (43%)	0.03
NM=3 (*n*=31)		28 (90.3%)	3 (9.7%)	
		**<Median**	**>Median**	
*Age (n=49)*				
⩽50 years (*n*=24)		2 (20%)	8 (80%)	0.04
>50 years (*n*=25)		22 (56%)	17 (44%)	
				
*Margin type (n=38)*				
Infiltrating (*n*=19)		13 (65%)	7 (35%)	0.05
Pushing (*n*=19)		6 (33%)	12 (67%)	
				
	**0**	**<Mean**	**>Mean**	
*Size (n=49)*				
Continuous variable				
Mean (s.d.)	54.83 (29.87)	37.19 (18.9)	22.33 (5.8)	0.02
				
*NPI (n=49)*				
Continuous variable				
Mean (s.d.)	5.76 (1.35)	5.12 (1.18)	3.94 (0.59)	0.03

IHC=immunohisto chemistry; NPI=Nottingham prognostic index; PRB=progesterone B.

**Table 3:** Significant variables associated with disease free interval on univariate analysis

**Variable**	***P*-value**	**Hazard ratio**
*Lymph node status*		
0	—	1
1–3	0.06	3.1
>3	0.02	3.8
		
*Lymph node status*		
Positive	0.02	3.5
Negative	—	1
		
*ERα immunohistochemistry*		
Positive	0.03	0.4
Negative	—	1
		
*Stage*		
I	—	1
II	0.09	2.7
III+IV	0.003	6
		
*CEA*		
Positive (>5)	0.01	4.3
Negative (⩽5)	—	1
		
*CA 15.3*		
Positive (>40)	0.05	4.3
Negative (⩽40)	—	1
		
*Gas6 mRNA*		
Above the mean	0.99	0
Below the mean	—	1
		
*Gas6 mRNA*		
Above the median	0.08	0.3
Below the median	—	1

**Table 4:** Significant variables associated with survival on univariate analysis

**Variable**	***P*-value**	**Hazard ratio**
*Lymph nodes*		
0	—	1
1–3	0.1	3.4
>3	0.01	7.1
		
*Lymph nodes*		
Positive compared to negative	0.03	5
		
NPI (Blamey, 1996)		
Continuous variable	0.003	1.9 per unit increase in NPI
		
*Stage*		
I	—	1
II	0.22	2.6
III+IV	0.002	11.4
		
*CA 15.3*		
Positive (>40)	0.05	11.4
Negative (⩽40)	—	1
		
*Size*		
>50 mm	0.04	2.9
<50 mm	—	1
		
*Margins*		
<10	0.05	0.13
>10	—	1
		
*Gas6 mRNA*		
Above the mean	0.99	0
Below the mean	—	1
		
*Gas6 mRNA*		
Above the median	0.15	0.36
Below the median	—	1

NPI=Nottingham prognostic index.
